# Molecular docking and dynamics simulation of main protease of SARS-CoV-2 with naproxen derivative

**DOI:** 10.6026/97320630019358

**Published:** 2023-04-30

**Authors:** Rageh K Hussein, Mohammad Marashdeh, Ahmed M. El-Khayatt

**Affiliations:** 1 Department of Physics, College of Science, Imam Mohammad Ibn Saud Islamic University (IMSIU), Riyadh, Saudi Arabia

**Keywords:** Naproxen derivative, SARS-CoV-2, drug-likeness and ADMET properties, Molecular docking, Molecular dynamics

## Abstract

Naproxen is a well-known anti-inflammatory drug that is frequently used to relieve inflammation, stiffness, and swelling. Naproxen has previously demonstrated antiviral activity, particularly against the influenza-A virus. There have been previous studies regarding naproxen effect on SARS-CoV-2 infection. Therefore, it is of interest to document the molecular docking and dynamics simulation data of main protease of SARS-CoV-2 with naproxen derivative for further consideration.

## Background:

The non-steroidal anti-inflammatory drug (NSAID) naproxen is the most popular derivative of propionic acid used to treat a variety of infections or symptoms. Naproxen has been used safely for many years due to its good cardiovascular characteristics [1, 2]. Currently, scientists are interested in the pharmacological properties of naproxen specifically its anticancer and antiviral properties [3, 4]. The analgesic and anti-inflammatory effects of naproxen are produced by preventing arachidonate binding, which inhibits both COX-1 and COX-2 cyclooxygenase isoenzymes in a competitive manner. Arachidonic acid is converted to prostaglandin G by COX-1 and COX-2 catalysts. Many cancer types infection, which are frequently characterized by COX-2 activity, may be avoided by inhibiting COX-2 enzymes [5, 6]. Many studies in the meantime are focused on the activities of new naproxen derivatives. *in-silico* investigation offers an efficient method for identifying and predicting the bioactivities of molecules, and provided a significant advancement in drug design and development [7, 8]. The *in-silico* approaches involve using computer-aided methods to increase the efficacy of known drugs or design new compounds. Computer-aided technologies or molecular modelling allows for virtual screening a wide range of compounds and their interactions with bio targets, which speeds up drug development with lower costs [9, 10]. Therefore, it is of interest to document the molecular docking and dynamics simulation data of main protease of SARS-CoV-2 with naproxen derivative for further consideration.

##  Material and Methods:

**Figure 1:**
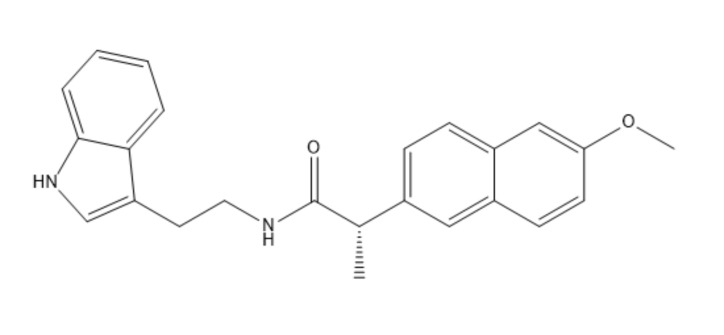
Molecular structure of N-(2-(1H-Indol-3-yl) ethyl)-2-(6-methoxynaphthalen-2-yl) propanamide compound

The proposed compound N-(2-(1H-Indol-3-yl) ethyl)-2-(6-methoxynaphthalen-2-yl)propanamide is a novel naproxen derivative (shown in Figure 1) synthesized and extensively characterized spectroscopically by Manolov *et al*. [11]. The physicochemical properties associated with the drug likeness character were calculated by using the online webserver Molinspiration (https://www.molinspiration.com/) and the OSIRIS property explorer code (https://www.organic-chemistry.org/prog/peo/). The integrated online service for ADMET properties prediction (ADMET Prediction Service) was used to assess a number of ADMET properties [12]. Molecular docking investigations were carried out using Autodock 4.2 software [13]. The main protease of SARS-CoV-2 was represented by the PDB: 6LU7 file, that was retrieved from the Protein Data Bank. The molecular dynamics simulation was done using the NAMD 2.13 software [14]. CHARMM General Force Field (CGenFF) was utilized to generate the parameter files and topology of the system. The equilibration of the system was obtained using two successive steps of the canonical isothermal-isochoric (NVT) ensemble and the isothermal-isobaric (NPT) ensemble. Using Periodic Boundary conditions, the simulation was run at a constant pressure of 1 atm and temperature of 310 K.

## Result and Discussion:

Lipinski's Rule is one of the earliest and best known rules, to evaluate physicochemical properties of drugs to determine whether they are consistent with the potential of effective oral absorption. According to Lipinski's Rule, the molecule's potential to display favorable drug-likeness character is determined by its molecular weight, the number of hydrogen-bond donors/acceptors (HBD/HBA) and octanol/water coefficient (logP) value [15]. Moreover, oral bioavailability is significantly correlated with the number of rotatable bonds (nrotb) and polar surface area (PSA) [16]. The calculated physicochemical parameters, obtained from Lipinski's rule of five, along with the associated criteria are listed in Table 1. The studied compound, as shown in the table, complied with the criteria for Lipinski's rule of five without any violations. The low polar surface area (PSA) value (54.12 Å²) results in maximum absorption and a sign of good oral bioavailability.

Also, in order to cut costs and time in drug development, it is crucial to assess the possible toxicity risk of a drug candidate. The toxicity level of mutagenicity, tumorigenicity, irritant, and reproductive was estimated using a color-coded methodology. Each toxicity parameter has a level represented by a red or green color. The red color indicating a high toxic risk and green indicating a low toxicity level. The results each toxicity category in Table 1 demonstrated a safe and minimal toxic behaviour for the studied compound.

**Figure 2:**
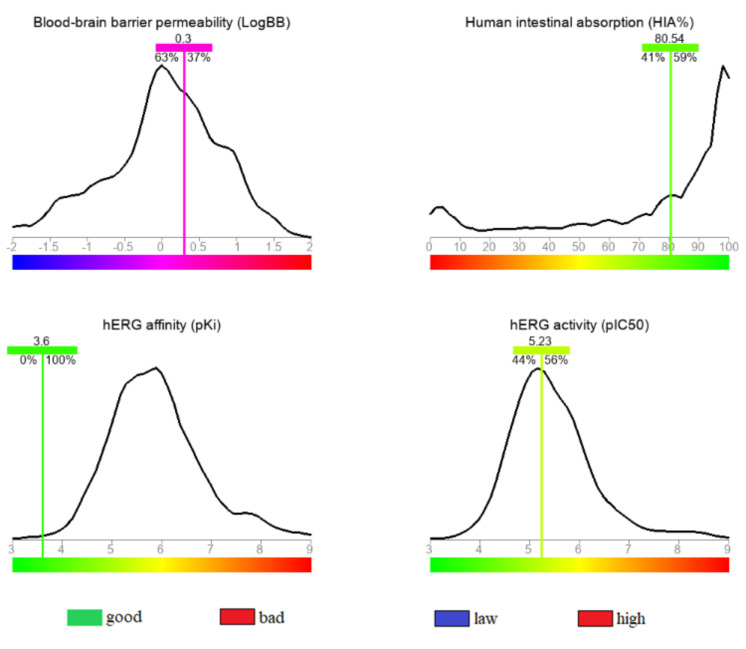
The estimated ADMET profile for the studied compound

Four ADMET properties, which are human intestinal absorbance (HIA), blood-brain barrier (BBB), hERG affinity (pki) and hERG activity, were calculated using a colored distribution chart demonstrating the predicted value and where it falls within a validated drug library. As shown in Figure 2, the measured human intestinal absorbance (HIA) value was 80.54% and was higher than 41% of the previously tested compounds. The standard limit of good human intestinal absorbance (HIA) value is 80% [17], and the measured value suggested a satisfactory oral absorption of the study compound. According to the blood-brain barrier (BBB) predictive model, a drug's ability to penetrate the central nervous system lies in the range of 0.3 to 1 [18]. The observed BBB value of 0.3 shows that the compound is at the minimum ability for entry to the central nervous system. The inhibition of the hERG by drugs or small molecules cause its disruption, which leads to diseases such as entricular arrhythmia.The hERG activity value ( pIC50 = 5.23) revealed a low inhibition level of the hERG channel. The determined hERG affinity value (pKi =3.6) classified the compound as safe in terms of hERG binding affinity.

**Figure 3:**
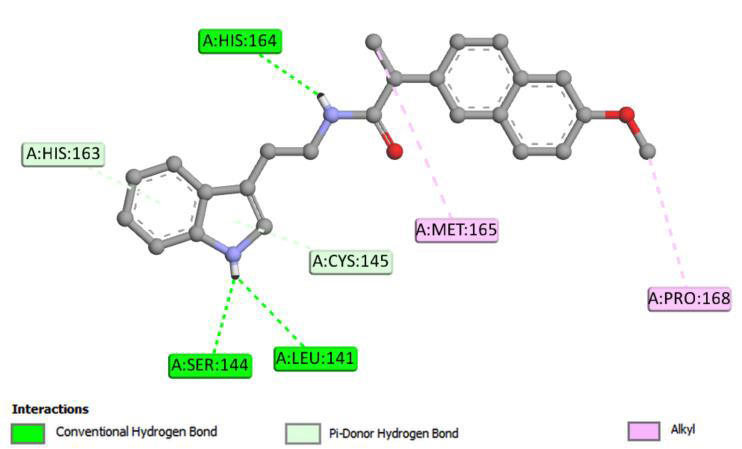
2D illustration of the most stable docking interaction of the proposed compound within the active site of 6lu7 main protease

Table 2 displays the results of docking the title compound into the active site pocket of 6lu7 Mpro. According to the results, the studied derivative revealed a strong binding affinity with a high binding energy of -9.36 kcal/mol. The Naproxen standard drug was evaluated using the same molecular docking procedure, and the binding energy was found to be -6.11 kcal/mol. Non-steroidal anti-inflammatory drugs (NSAIDs) including Naproxen were studied as potential inhibitors for the main SARS-CoV-2 protease in recent study. Of these drugs, Talniflumat has the lowest binding energy (- 8.7 kcal/mol), whereas Naproxen scored - 6.3 kcal/mol [19]. Another earlier study repurposed a set of NSAIDs approved drugs to make use of an efficient SARS-CoV-2 treatment, the best binding affinity was revealed by Sulfinpyrazone -7.12 kcal/mol [20]. Our results demonstrated that the proposed compound was successful in binding to 6lu7 Mpro with the most considerably minimum binding energy. The extremely low value of the inhibition constant value, 0.00137 µM, was also observed as evidence of the high binding affinity. The best binding conformation for the interaction of the ligand with the target protein is depicted in Figure 3. The protein-ligand complex was stabilized by hydrogen bonding, pi-donor hydrogen bonding and Alkyl interactions. The hydrogen bonding interaction is another indicator of a high affinity. Three conventional hydrogen bonds with medium bond length of 2.83, 2.92 and 2.73 Å were formed with His164, Ser144 and Leu141 respectively. The indole ring of the ligand had two pi-donor hydrogen bonds with residues His 163 and Cys 145 with 3.41and 3.70 Å in length. Met165 and Pro168 amino residues were bonded to the terminal carbon atoms of ethyl and methoxynaphthalen groups, respectively, by an alkyl interaction with the bond lengths of 3.89 and 4.06 Å.

**Figure 4:**
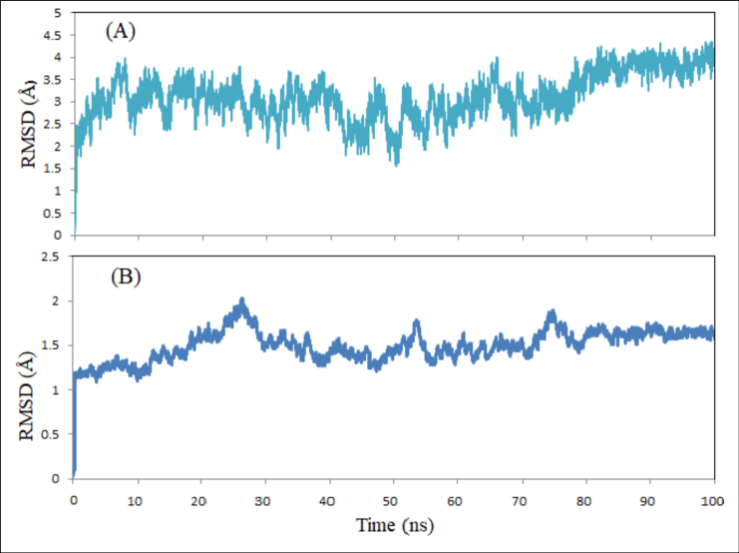
The RMSD graphs of the protein-ligand complex (A) and ligand (B) during 100 ns simulation.

The stability of the protein-ligand complex was investigated using root mean-square deviation (RMSD) analysis of the molecular dynamics trajectory over 100 ns. Figure 4 illustrates the calculated RMSD for the conformational variation of the protein-ligand complex and ligand. The RMSD of the protein and ligand plot indicated that there were no major deviations over the simulation time. Over the first 80 ns, the RMSD of the protein backbone fluctuated between 2 and 3.5 Å, then increased to ~ 4 Å and maintained stability until the simulation end. The ligand displayed behavior similar to that of the protein with lower RMSD values. The RMSD values of the ligand in the last 20 ns remained within a clearly stable range of 1.6 Å. This results confirmed that binding of the ligand in the active site of the protein remained stable throughout the molecular dynamics simulation.

##  Conclusion:

We document the molecular docking and dynamics simulation data of main protease of SARS-CoV-2 with naproxen derivative for further consideration.

## Figures and Tables

**Table 1:** The drug-likeness analysis of Lipinski's rule parameters, TPSA, nrotb, and the predicted toxicity profile.

**Drug-likeness**					
**MW (g/mol)**	**HBD**	**HBA**	**cLogP**	**nrotb**	**PSA(Å²)**
< 500	≤5	≤10	≤5	≤10	<140
372	2	4	4.54	7	54.12

**Table 2:** Docking results of the proposed compound against 6lu7 main protease of SARS-CoV-2

**Targe protein**	**Binding Energy (kcal/mol) **	**Inhibition Constant (µM)**	**Type of interaction**	**Interacting Residues**	**Bond Distance (Å)**
6lu7	− 9.36	0.00137	Hydrogen Bond	His164	2.83
			Hydrogen Bond	Ser144	2.92
			Hydrogen Bond	Leu141	2.73
			pi-donor hydrogen bond	His163	3.41
			pi-donor hydrogen bond Alkyl	Cys145	3.7
			Alkyl	Met165	3.89
				Pro168	4.06
